# Effect of Luanbai Glaze on the Coloration of Cobalt Pigment in Yuan Dynasty Jingdezhen Porcelains: An Experimental Study

**DOI:** 10.3390/ma19112254

**Published:** 2026-05-26

**Authors:** Jun Sun, Qijiang Li, Xiaoyan Xia, Min Tang, Yan Liang, Linxin Ouyang

**Affiliations:** 1 Research Center of Ancient Ceramic, Jingdezhen Ceramic University, Jingdezhen 333001, China; 2010024004@stu.jcu.edu.cn (J.S.); 192025@jcu.edu.cn (X.X.); tangmin@jcu.edu.cn (M.T.); 2 Jiangxi Ceramic Heritage Conservation and Imperial Kiln Research Collaborative Innovation Center, Jingdezhen 333001, China; 3 Hunan Provincial Key Laboratory of Intelligent Protection and Utilization Technology in Masonry Artifacts, Hunan University of Science and Engineering, Yongzhou 425199, China; yanliang1022@126.com; 4 Handicraft Art College, Hunan Institute of Art and Design, Yiyang 413000, China; 041029@jcu.edu.cn

**Keywords:** Luanbai glaze, blue-and-white porcelain, cobalt pigment, technological compatibility, refiring experiment

## Abstract

This study investigates whether Yuan Dynasty Jingdezhen Luanbai glaze can support cobalt-blue coloration under conditions relevant to early blue-and-white porcelain production. Comparative analyses of archaeological Luanbai and blue-and-white specimens show that the two glaze types have similar average thicknesses, approximately 0.20 mm, and comparable basic chemical compositions, especially in their SiO_2_ and Al_2_O_3_ contents. These results suggest that they belong to related high-temperature calcium–alkali glaze traditions rather than completely isolated technological systems. Simulated firing experiments using five cobalt pigments of different compositions further indicate that Luanbai glaze can support cobalt-blue coloration at 1300 °C in a reducing atmosphere. Compared with unglazed controls, Luanbai-glazed samples showed a more consistent blue appearance and clearer pigment–glaze interaction. XPS and SEM-EDS line-scan analyses revealed differences in the near-surface chemical environment and cross-sectional distribution of Co, Mn, and Fe between glazed and unglazed samples, supporting the role of glaze coverage in color development. Refiring experiments on authentic Yuan sherds further supported the feasibility of cobalt-blue coloration on historical Luanbai glaze surfaces. Overall, the results suggest that the opalescent appearance of Luanbai glaze is not an inherent barrier to underglaze cobalt decoration. This work provides experimental evidence for reassessing the technological relationship between Luanbai and early blue-and-white porcelain.

## 1. Introduction

Jingdezhen, located in Jiangxi Province, China, was one of the most important ceramic production centers in the history of Chinese porcelain [1,2]. From the Song to Yuan dynasties, Jingdezhen kilns produced a variety of high-fired porcelain wares, among which Qingbai porcelain, Luanbai porcelain, and blue-and-white porcelain were particularly representative [3,4]. Qingbai porcelain is generally characterized by a transparent or semi-transparent bluish-white glaze. Luanbai porcelain, also known as egg-white glazed porcelain, is distinguished by a warm white glaze with a slight bluish tone and a certain degree of opalescence or opacity [5]. Blue-and-white porcelain, by contrast, is known for cobalt-blue motifs painted beneath a transparent glaze and fired at high temperature [6,7]. These ceramic types represent important stages in the technological development of Jingdezhen porcelain during the Song–Yuan period.

The Yuan Dynasty was a crucial period for the development of Jingdezhen ceramic technology. Both Luanbai porcelain and blue-and-white porcelain emerged and developed during this period, but they display markedly different visual appearances: Luanbai glaze appears soft, warm, and opalescent, whereas the white ground of blue-and-white porcelain is usually more transparent and provides a clear background for cobalt-blue decoration. Because of this optical contrast, the two wares have often been regarded as products of different technological and aesthetic traditions. However, from a materials and processing perspective, visual differences do not necessarily imply completely separated technological systems. Previous archaeometric studies have shown that Qingbai porcelain, Luanbai porcelain, and blue-and-white porcelain may share relationships in raw material selection, glaze preparation, firing practice, and high-temperature glaze systems [8,9]. In particular, studies of Luanbai and Qingbai glazes from Hutian kiln indicate that Luanbai glaze developed within the broader Song–Yuan Qingbai-glazed porcelain tradition [1]. Its opalescent appearance is closely related to glaze composition, firing temperature, cooling process, and microstructural development, rather than to an entirely independent glaze tradition [1,5].

Research on historical blue-and-white porcelain has shown that cobalt-blue coloration is a complex process and is not controlled solely by the bulk composition of the cobalt pigment [10,11,12]. The final color can be affected by cobalt source and composition, associated elements such as Mn and Fe, pigment particle distribution, Co speciation, Co-bearing crystalline phases, glaze color, firing atmosphere, and pigment–glaze interaction [13,14]. Studies on the bleeding effect of cobalt pigments further suggest that cobalt migration in vitreous silicate should be understood as a diffusion-related process, while microstructural features such as anorthite or Co-bearing spinel phases may influence pigment dissolution, diffusion, and color dispersion [15,16,17,18]. Therefore, whether a glaze appears transparent or opalescent after firing should not be directly equated with whether it can provide a suitable high-temperature glaze environment for cobalt-blue coloration. However, the specific question of whether Yuan Dynasty Jingdezhen Luanbai glaze can support cobalt-blue coloration has not been directly tested. This issue is important because it concerns not only the material compatibility between Luanbai glaze and cobalt pigment, but also the broader technological relationship between Yuan Luanbai porcelain and early blue-and-white porcelain.

Based on this premise, this study focuses on Yuan Dynasty Luanbai and blue-and-white porcelain specimens from the Museum of Jingdezhen Ceramic University. By comparing glaze thickness, body and glaze chemical compositions, and the colorimetric characteristics of the white-ground and blue-decorated areas, and by combining simulated firing experiments, X-ray photoelectron spectroscopy (XPS), SEM-EDS line-scan analysis, and refiring experiments on historical specimens, this paper investigates the material and technological relationship between Yuan Dynasty Jingdezhen Luanbai glaze and cobalt-blue coloration [19,20]. Specifically, this study addresses three main issues: first, the technical comparability of Luanbai glaze and blue-and-white glaze in terms of their material foundations; second, the ability of Luanbai glaze to support cobalt-blue coloration under a high-temperature reducing atmosphere; and third, the further assessment of this compatibility through refiring experiments on authentic Yuan Dynasty specimens.

## 2. Materials and Methods

### 2.1. Sample Sources

**Figure 1:**
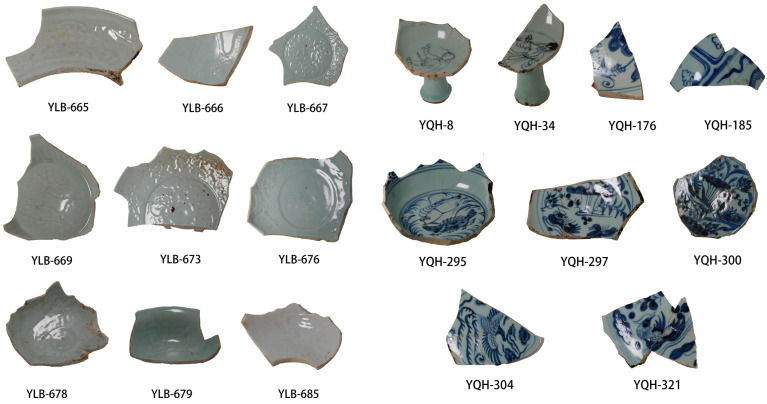
Yuan Dynasty Luanbai and blue-and-white porcelain test specimens used in this study.

The samples used in this study consisted of two parts: archaeological specimens from Yuan Dynasty Jingdezhen and experimentally prepared samples. A total of 18 archaeological specimens were provided by the Museum of Jingdezhen Ceramic University, including 9 Luanbai porcelain specimens and 9 blue-and-white porcelain specimens (Figure 1). The Luanbai porcelain samples were labeled as the YLB series, and the blue-and-white samples were labeled as the YQH series. The Luanbai specimens featured a warm, soft white tone overall, with slight bluish characteristics locally and a certain opalescent opacity. The blue-and-white specimens exhibited a sharp visual contrast between the white ground and the decorated areas, with the white ground typically showing high transparency and a “bluish-white” effect, while the blue motifs ranged from light blue and grayish-blue to deep blue.

In addition to the 18 archaeological specimens, 2 small historical Yuan Dynasty fragments (1 Luanbai and 1 blue-and-white) were selected for refiring experiments. Both retained their original historical glaze surfaces and were used to evaluate the coloration performance of experimental cobalt pigments on authentic Yuan Dynasty glazes and their interaction with the glaze layer. Thus, the sample system in this paper can be divided into three categories: archaeological specimens for material testing, experimental samples for simulated firing, and historical specimens for refiring verification.

### 2.2. Analytical Methods

To systematically compare the material characteristics of Yuan Dynasty Luanbai and blue-and-white glazes, glaze thickness, chemical composition, and colorimetry were measured on the archaeological specimens. In addition, XPS analysis was performed on representative experimental samples to evaluate the influence of glaze coverage on the surface chemical environment of the cobalt pigment.

The cross-sections of the glazes were observed using a VHX6000 digital microscope (Keyence Corporation, Osaka, Japan) to record the morphology and structural characteristics. Glaze thickness was measured using an optical coherence tomography (OCT) system (Thorlabs Inc., Newton, NJ, USA) in 2D scanning mode. This method was primarily used to compare the macroscopic glazing differences between the two porcelain types, providing structural context for discussing the influence of the glaze environment on pigment–glaze interaction.

The chemical compositions of the bodies and glazes were analyzed using an Eagle III XXL energy-dispersive X-ray fluorescence (EDXRF) spectrometer (EDAX Inc., Mahwah, NJ, USA). The comparison of body compositions helped determine whether the two types shared similar paste preparation technologies. The glaze composition comparison analyzed the similarities and differences in the basic glass network and fluxing systems, establishing the material basis for assessing whether Luanbai glaze could support cobalt-blue coloration [21,22].

Surface colorimetry was conducted using an NF-333 portable spectrophotometer (Nippon Denshoku Industries Co., Ltd., Tokyo, Japan). The test areas included the white grounds of both porcelain types and the blue motifs of the Yuan blue-and-white specimens. The colorimetric data of the white grounds were used to compare the overall color perception differences, while the data for the blue areas quantified the comprehensive color characteristics of Yuan Dynasty cobalt motifs.

To assist in understanding the effect of glaze coverage on the coloration environment, XPS analysis was performed using a K-Alpha X-ray photoelectron spectrometer (Thermo Fisher Scientific, East Grinstead, UK) on two types of experimental blue-decorated areas: one painted directly on the biscuit and fired without glaze, and the other fired with a Luanbai glaze coating. The analysis focused on the signals of Fe, Co, and Mn to compare differences in the surface chemical environment before and after glaze coverage.

To obtain further information on cross-sectional microstructure and elemental distribution, representative samples from the simulated firing experiments and refiring experiments were examined using a Prisma E scanning electron microscope equipped with a tungsten filament (Thermo Fisher Scientific, Brno, Czech Republic). Energy-dispersive X-ray spectroscopy (EDS) line-scan analyses were also conducted across the glaze layer and the pigment–glaze region. The analysis focused on the distribution characteristics of Fe, Co, and Mn within the glaze layer, pigment zone, and adjacent interfacial areas, in order to evaluate the interaction between the cobalt pigment and the glaze as well as possible elemental migration trends under different glaze conditions. These results complement the XPS analysis and provide microstructural and elemental-distribution evidence for discussing the influence of glaze coverage on the cobalt-blue coloration environment.

### 2.3. Simulated Firing Experiments

To verify the compatibility of Luanbai glaze with cobalt-blue coloration, simulated firing experiments were designed. The Luanbai and blue-and-white glaze recipes were formulated based on the chemical compositions of the archaeological specimens. Raw materials included Yaoli glaze stone, glaze ash, and potassium feldspar. The materials were mixed according to the set ratios and wet-milled with a mass ratio of *m*_material_:*m*_ball_:*m*_water_ = 1.0:2.0:0.6. The milling speed was 400 r/min for 20 min. After sieving, the glaze was applied via the dipping method.

Jingdezhen modern high-temperature porcelain clay was used uniformly for the experimental bodies, pressed into 6 cm diameter test pieces to minimize body-related interference. The main oxide compositions of the two experimental glazes are shown in Table 1.

The firing experiments were carried out in a 0.2 m^3^ shuttle gas kiln using liquefied petroleum gas, mainly composed of propane, as the fuel. The firing schedule was as follows: the samples were first heated to 980 °C under an oxidizing atmosphere over 5.5 h and then held at 980 °C for 0.5 h. The kiln atmosphere was subsequently changed to a reducing atmosphere, with the CO concentration controlled at approximately 3–4%, and the temperature was further increased to 1300 °C over 3 h. After reaching the maximum temperature, the kiln was shut down and the samples were naturally cooled to room temperature. This firing schedule was designed to simulate, as closely as possible, the reaction process between the cobalt pigment and the glaze layer under high-temperature reducing conditions.

Five cobalt pigment raw materials with different compositional characteristics were selected and labeled qh01–qh05. Their chemical compositions differ significantly (Table 2), covering varying contents of MnO, Fe_2_O_3_, and CoO, making them suitable for comparing the coloration behavior of different cobalt pigments under different glaze conditions. During the experiment, an appropriate amount of water was added to the cobalt pigment powder, and the mixture was homogenized with a brush before being painted onto the surface of the ceramic body. The glaze was applied by dipping, with the glaze thickness controlled at approximately 0.2 mm. According to the glaze conditions, the experimental samples were divided into three groups: the “Luanbai glaze + cobalt pigment” group, the “blue-and-white glaze + cobalt pigment” group, and the unglazed control group. All three groups were fired under the same firing schedule. After firing, the overall color, coloration stability, and interaction between the pigment and the glaze layer in the blue-decorated areas were observed, and the color characteristics of these areas were quantitatively evaluated using L*, a*, and b* values.

### 2.4. Refiring Experiments on Historical Specimens

Building upon the simulated firing experiments, this study conducted refiring experiments on authentic Yuan Dynasty specimens to further assess the applicability of the experimental results to actual historical glazes. Two small authentic Yuan Dynasty specimens were selected for the refiring experiments: one Yuan Dynasty Luanbai porcelain specimen and one Yuan Dynasty blue-and-white porcelain specimen. In the experiments, cobalt pigment was applied onto the original glaze surfaces of both specimens, followed by refiring at 1300 °C under a reducing atmosphere. The firing curve was identical to that of the simulated experiments.

The primary observational focuses of the refiring experiments included two aspects: first, whether the cobalt pigment could develop cobalt-blue coloration on authentic Yuan Dynasty glazes; and second, whether the fired cobalt pigment could interact with or bond to the original glaze layer. After firing, macroscopic observations were conducted on the blue coloration areas of both types of samples, and their average L*, a*, and b* values were measured to compare the overall color performance of the cobalt pigment on the authentic glazes of the Yuan Dynasty Luanbai and blue-and-white porcelains.

## 3. Results and Discussion

### 3.1. Material Basis Comparison of Yuan Dynasty Luanbai and Blue-And-White Porcelains

#### 3.1.1. Glaze Thickness and Cross-Sectional Structural Characteristics

**Figure 2:**
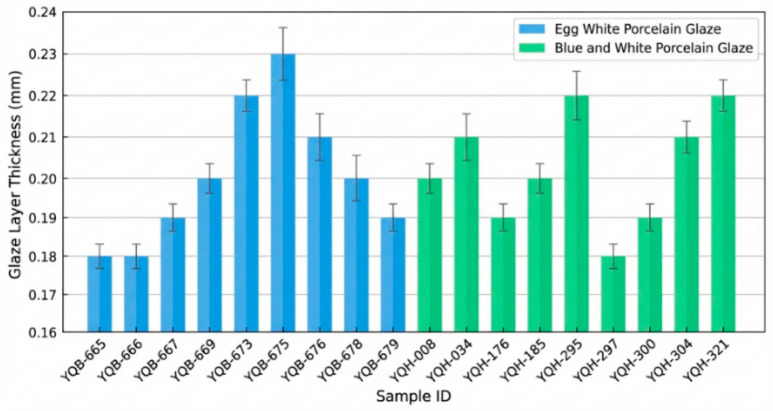
Glaze thicknesses of Yuan Luanbai and blue-and-white porcelains.

OCT measurements indicated that the glaze thicknesses of the two types of samples were generally similar (Figure 2). Luanbai samples ranged from 0.18–0.23 mm (average 0.20 ± 0.02 mm), while blue-and-white samples ranged from 0.18–0.22 mm (average 0.20 ± 0.01 mm). The near-identical average thickness demonstrates that the two glazes are highly comparable at the application scale. Technologically, this suggests that the thickness of Luanbai glaze does not constitute an inherent obstacle to cobalt-blue coloration [23].

**Figure 3:**
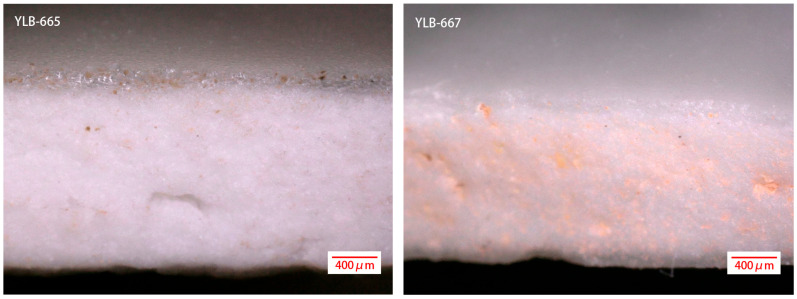
Cross-sections of the glaze layers of YLB-665 and YLB-667.

**Figure 4:**
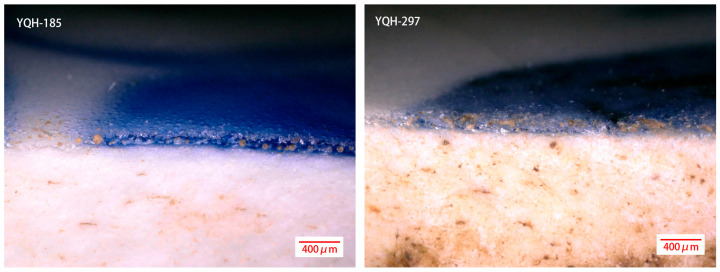
Cross-sections of the glaze layers of YQH-185 and YQH-297.

Microscopic observation of cross-sections further revealed that both glazes were continuous and bonded well with the body without severe delamination. The Luanbai glaze exhibited a uniform opalescence (Figure 3), while the blue-and-white glaze was more transparent (Figure 4). Although their final optical effects differ significantly, this variance is not due to thickness but is more likely related to composition, melting state, and microstructure. Therefore, the opalescent appearance alone is insufficient to conclude that Luanbai glaze cannot serve as a medium for cobalt coloration.

#### 3.1.2. Chemical Composition of Body and Glaze and Technical Continuity

Chemical analysis (Table 3) suggests that the two porcelain types share related material characteristics [1,17,24]. The average composition of Luanbai glaze was SiO_2_ 72.74 wt.%, Al_2_O_3_ 13.42 wt.%, CaO 4.74 wt.%, MgO 0.22 wt.%, K_2_O 3.67 wt.%, Na_2_O 3.31 wt.%; while blue-and-white glaze averaged SiO_2_ 72.67 wt.%, Al_2_O_3_ 12.48 wt.%, CaO 6.30 wt.%, MgO 0.42 wt.%, K_2_O 3.50 wt.%, Na_2_O 2.63 wt.%. The nearly identical SiO_2_ and Al_2_O_3_ contents indicate a consistent basic glass network. The primary differences lay in the fluxing components (alkali and alkaline earth metals), with blue-and-white glaze having slightly higher CaO and MgO, and Luanbai having slightly higher K_2_O and Na_2_O.

This result indicates that Luanbai and blue-and-white glazes were not based on two completely distinct glaze systems. Rather, they appear to have achieved different final glaze effects through adjustments in flux proportions within related high-temperature porcelain glaze systems. In other words, the two show a degree of technological continuity in their material foundations, rather than being mutually isolated material systems [5,25]. For this study, this point is particularly significant, as it demonstrates that Luanbai glaze, at least in terms of material composition, does not present a fundamental barrier to cobalt-blue coloration.

The comparison of the body compositions also supports this conclusion (Table 4). Both types of samples exhibit the characteristics of high-temperature porcelain bodies. Although individual specimens show certain fluctuations in SiO_2_, Al_2_O_3_, and impurity components, overall, there is no indication that they belong to two completely independent body-manufacturing systems. Therefore, subsequent discussions regarding whether Luanbai glaze can support the cobalt-blue coloration of the underglaze blue should focus on the glaze environment and the firing reaction mechanisms, rather than on the differences in the porcelain bodies.

#### 3.1.3. Colorimetric Characteristics of the White Ground and Cobalt Blue Areas

The colorimetric test results show (Table 5) that there are indeed differences in the visual appearance of the white ground areas between Luanbai and blue-and-white porcelains [26]. The average colorimetric values for the white ground of Luanbai porcelain are L* = 70.43 ± 3.35, a* = −3.19 ± 1.05, and b* = 2.55 ± 0.90; whereas the average values for the white ground of blue-and-white porcelain are L* = 67.95 ± 3.64, a* = −3.92 ± 0.41, and b* = 3.27 ± 1.85. Overall, the white ground of Luanbai porcelain exhibits higher lightness and a softer overall color perception. In contrast, the white ground of blue-and-white porcelain presents a more negative a* value, an overall hue that leans more towards a bluish-green tone, and greater fluctuation in b* values, with some samples displaying a relatively distinct characteristic of “white with a bluish tinge.” This is consistent with the macroscopic visual perception of the glaze surfaces of the two types of wares.

However, this difference primarily reflects variations in the optical performance of the fired finished products, and should not be directly equated with a technological judgment regarding its “suitability for blue-and-white coloration.” In other words, the fact that the white ground of Luanbai glaze is relatively soft and possesses an opalescent characteristic does not mean that it cannot provide a suitable high-temperature glaze environment for the cobalt pigment during firing. What the colorimetric differences of the white ground reveal is a variance in the visual effect of the glaze surface, rather than a fundamental opposition in coloration conditions.

The colorimetric results of the blue-and-white decorated areas on the Yuan Dynasty blue-and-white specimens further show (Table 6) that the overall color distribution across different samples is relatively broad, but generally exhibits lower L* values and distinctly negative b* values, indicating that Yuan Dynasty blue-and-white decorations generally show a clear blue tendency overall. The significant differences in overall color among various specimens also suggest that the final coloration of the underglaze blue is not determined solely by the cobalt pigment itself, but is closely related to the glaze background, melting state, and firing regime [10,27]. Thus, it is evident that the glaze environment itself is a crucial factor influencing the overall color performance of the blue-and-white decoration.

#### 3.1.4. XPS Results and the Effect of Glaze Coverage

**Figure 5:**
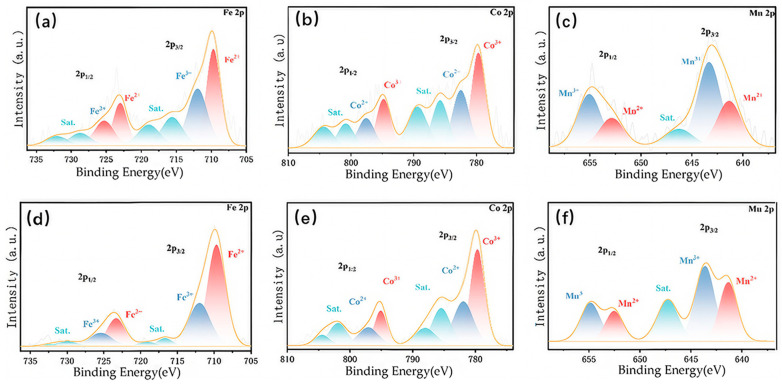
XPS spectra of the blue-colored areas after firing qh05 under Luanbai-glaze-covered and unglazed conditions. (**a**) Luanbai glaze–Fe 2p; (**b**) Luanbai glaze–Co 2p; (**c**) Luanbai glaze–Mn 2p; (**d**) unglazed–Fe 2p; (**e**) unglazed–Co 2p; (**f**) unglazed–Mn 2p. The colored peaks represent the deconvoluted fitting components assigned to the corresponding chemical states and satellite peaks.

To further analyze the influence of glaze coverage on the coloration environment of cobalt pigment, qh05 was selected as a representative cobalt pigment for high-resolution X-ray photoelectron spectroscopy (XPS) analysis. The blue-colored areas of the Luanbai-glazed sample and the unglazed sample were analyzed, and the results are shown in Figure 5. The analysis focused on Co, Mn, and Fe, which are closely related to cobalt-blue coloration.

The Co 2p spectra show that cobalt in both samples existed in mixed valence states, with Co^2+^ and Co^3+^ signals accompanied by satellite peaks [28]. This indicates that cobalt was present in a complex chemical environment after firing. Compared with the unglazed sample, the Co 2p spectra of the Luanbai-glazed sample show clear differences, which is consistent with its stronger blue appearance. The corresponding colorimetric values also show this contrast: the Luanbai-glazed qh05 sample had L* = 42, a* = 3, and b* = −24, whereas the unglazed qh05 sample had L* = 35, a* = 0, and b* = −3. This suggests that the blue appearance was much weaker under unglazed conditions.

The Mn 2p and Fe 2p spectra also indicate mixed chemical states in both samples. Mn and Fe are associated components of cobalt pigments and may influence the hue, darkness, and overall visual appearance of the final color [29,30]. In the unglazed sample, the influence of these elements may be more directly expressed because the pigment particles were exposed without a continuous glaze layer. In contrast, in the Luanbai-glazed sample, glaze coverage was associated with a different near-surface chemical environment for Co, Mn, and Fe.

Overall, the XPS results suggest that glaze coverage is related to changes in the near-surface chemical environment of the cobalt pigment after firing. These surface-chemical differences are consistent with the stronger cobalt-blue appearance observed in the Luanbai-glazed sample. However, because XPS is mainly sensitive to the near-surface region, these results should be regarded as complementary evidence for surface chemical changes rather than direct proof of the complete pigment–glaze interfacial reaction mechanism.

### 3.2. Results of Simulated Firing Experiments and Their Significance

**Figure 6:**
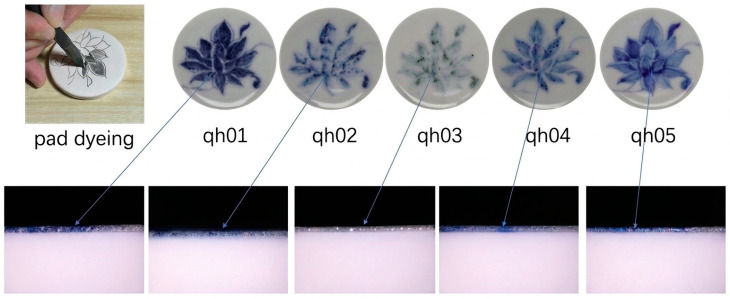
Color development of five cobalt pigments fired at 1300 °C in a reducing atmosphere under Luanbai glaze.

In the “Luanbai glaze + cobalt pigment” group (Figure 6), all five cobalt pigments developed cobalt-blue coloration to varying degrees after firing, and the pigment-bearing areas showed visible interaction with the glaze layer. Their average L*, a*, and b* values are presented in Table 7. The b* values of all five samples were negative, and most of them showed relatively large negative b* values, indicating that Luanbai glaze can support cobalt-blue coloration under the tested firing conditions. Among them, qh03 showed only a weak negative b* value, suggesting that differences in cobalt pigment composition still affected the depth and hue of the final color. Nevertheless, this does not change the experimental observation that Luanbai glaze can support cobalt-blue coloration under high-temperature reducing conditions. Although Luanbai glaze has an opalescent and relatively opaque appearance after firing, it can still provide a favorable high-temperature glaze environment for cobalt-blue color development.

**Figure 7:**
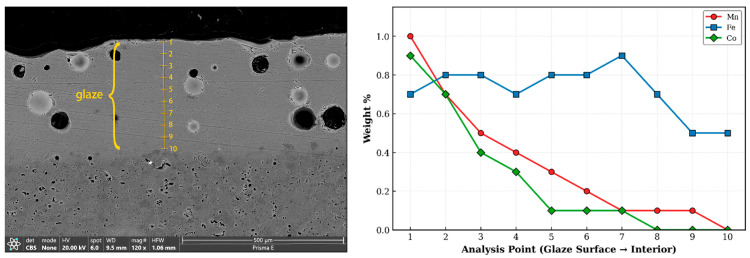
Cross-sectional SEM image and EDS line-scan profiles of the qh05 cobalt pigment fired under Luanbai-glaze coverage at 1300 °C in a reducing atmosphere. The numbered points indicate the EDS analysis positions from the glaze surface toward the interior; Mn, Fe, and Co contents are plotted as weight percentages. The colored curves in the EDS profiles represent the elemental contents indicated in the corresponding legends.

The cross-sectional SEM image of the Luanbai-glazed qh05 sample shows a continuous glaze layer with a generally compact glassy matrix and local pores. No obvious separation between the glaze layer and the underlying body can be observed (Figure 7). The EDS line-scan profile from the glaze surface toward the interior shows that Mn and Co are enriched near the upper glaze region and decrease gradually with increasing depth. Co decreases from the surface region to nearly zero in the deeper part of the profile, while Mn shows a similar downward trend. Fe remains detectable through a wider part of the glaze profile, which may reflect both the pigment contribution and the background composition of the glaze. This surface-to-interior gradient indicates that the cobalt pigment did not remain as a simple surface deposit, but may have been partly incorporated into or interacted with the molten Luanbai glaze during firing. Therefore, the SEM-EDS result provides cross-sectional support for the colorimetric observation that Luanbai glaze can provide a favorable glaze environment for cobalt-blue coloration under the tested firing conditions.

**Figure 8:**
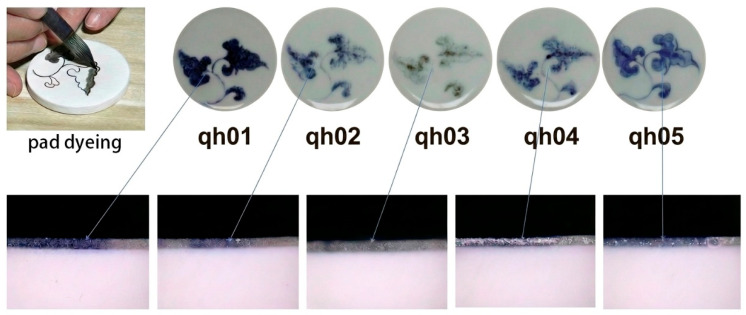
Color development of five cobalt pigments fired at 1300 °C in a reducing atmosphere under blue-and-white glaze.

In the “blue-and-white glaze + cobalt pigment” group (Figure 8), the five cobalt pigments were also able to interact with the glaze layer after firing and showed clear coloration differences. Their average L*, a*, and b* values are presented in Table 8. Most samples exhibited a blue tendency dominated by negative b* values, with qh04 and qh05 showing particularly strong blue appearances. However, qh03 showed a positive b* value, indicating that its final color deviated from a typical blue hue. This result shows that even under blue-and-white glaze conditions, the intrinsic composition of the cobalt pigment strongly affects the final color. Therefore, the glaze layer provides an important high-temperature environment for cobalt-blue color development, while the specific hue and intensity are further controlled by pigment composition.

**Figure 9:**
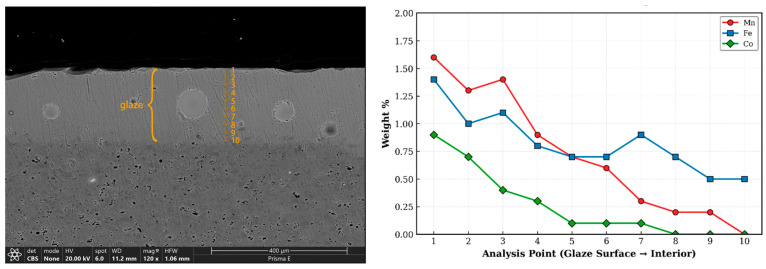
Cross-sectional SEM image and EDS line-scan profiles of the qh05 cobalt pigment fired under blue-and-white glaze coverage at 1300 °C in a reducing atmosphere. The numbered points correspond to the EDS analysis positions from the glaze surface toward the interior; Mn, Fe, and Co contents are plotted as weight percentages. The colored curves in the EDS profiles represent the elemental contents indicated in the corresponding legends.

The blue-and-white-glazed qh05 sample displays a continuous glaze layer with visible pores or bubbles, but the pigment-bearing region remains integrated with the glaze (Figure 9). The EDS line-scan data show that Mn and Co are concentrated near the glaze surface and decrease toward the interior. This trend is broadly comparable to that observed in the Luanbai-glazed sample. The Fe profile is relatively more persistent and fluctuates within the glaze, which is consistent with the presence of Fe both as an impurity in the pigment and as a minor component of the glaze matrix. The similarity between the blue-and-white glaze group and the Luanbai glaze group suggests that both glaze systems can provide a high-temperature glassy environment for pigment-glaze interaction. The result supports the argument that the visual opacity of Luanbai glaze after firing does not necessarily prevent cobalt pigment from integrating with the glaze during firing.

**Figure 10:**
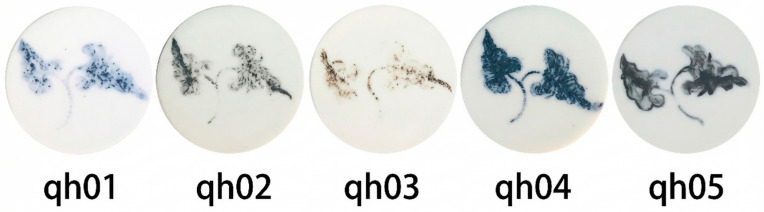
Color development of the unglazed control samples fired at 1300 °C in a reducing atmosphere.

Compared to the two aforementioned groups, the coloration characteristics of the unglazed control group are markedly different (Figure 10). The average L*, a*, and b* values of the five cobalt pigments fired under unglazed conditions are presented in Table 9. Observing the b* values, only qh01 and qh04 exhibited relatively distinct negative values, qh05 presented only a weak negative value, qh02 was close to neutral, and qh03 shifted to a distinctly positive value. Concurrently, the L* values across the samples fluctuated significantly, and the dispersion of the overall color increased notably. This indicates that in the absence of glaze coverage, although individual cobalt pigment samples may still retain a certain blue tendency, the overall coloration stability is poor, making it difficult to achieve a typical and uniform underglaze blue effect [31]. In other words, coloration under unglazed conditions does not imply that blue cannot appear at all; rather, the overall color output is significantly unstable and more prone to deviating towards grayish, pale, or abnormal hues.

**Figure 11:**
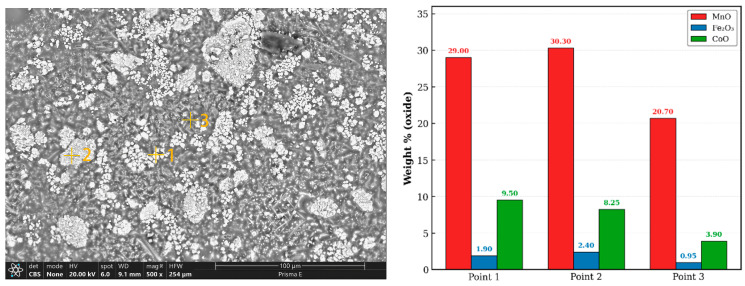
Surface SEM image and EDS point-analysis results of the unglazed qh05 cobalt pigment after firing at 1300 °C in a reducing atmosphere. The labeled points indicate selected pigment-rich areas analyzed by EDS; MnO, Fe_2_O_3_, and CoO contents are presented as weight percentages. The colored bars in the EDS chart represent the oxide contents obtained from EDS point analysis.

The unglazed qh05 sample differs clearly from the two glaze-covered samples. The SEM surface image shows a heterogeneous distribution of pigment particles and particle aggregates rather than a continuous glassy coverage (Figure 11). EDS point analyses indicate high but uneven contents of MnO and CoO in selected pigment-rich areas, while Fe_2_O_3_ remains relatively low. The large compositional differences among the measured points reflect the heterogeneous nature of the exposed pigment layer. In the absence of glaze coverage, the cobalt-bearing particles are not incorporated into a continuous silicate glaze matrix, and no surface-to-interior elemental gradient comparable to the glazed samples can be established. This explains why the unglazed group shows weaker and less consistent cobalt-blue coloration: the presence of cobalt pigment alone is not sufficient to produce a uniform underglaze-blue effect without an appropriate glaze environment.

To further evaluate the colorimetric relationship between the experimental samples and archaeological Yuan blue-and-white motifs, the L*, a*, and b* values of the experimental groups were compared with those of the blue-decorated areas of the Yuan blue-and-white specimens listed in Table 10. The color difference was calculated as ΔE*ab = [(ΔL*)^2^ + (Δa*)^2^ + (Δb*)^2^]^1/2^, using the mean L*, a*, and b* values of the archaeological Yuan blue-and-white motifs as the reference. The archaeological blue motifs show a relatively broad colorimetric distribution, indicating that “Yuan blue” should not be understood as a single fixed color value. Therefore, in this study, cobalt-blue coloration was evaluated by combining visual observation, negative b* values, and colorimetric proximity to the archaeological reference mean. The calculated ΔE*ab values show that the Luanbai-glazed group and the refired Luanbai sample are closer to the archaeological reference mean than the unglazed group, although differences in lightness and hue remain among different pigments. These results support the interpretation that Luanbai glaze can provide a favorable glaze environment for cobalt-blue coloration, while the specific hue and intensity are still controlled by pigment composition and firing-related factors.

It should be noted that ΔE*ab values describe colorimetric distance only and should be interpreted together with visual observation and SEM-EDS evidence for pigment–glaze interaction.

The comparative results of the three groups indicate that the glaze environment exerts an important influence on the coloration of cobalt pigments [13,18]. Under Luanbai glaze coverage, all five cobalt pigments showed blue appearances dominated by negative b* values, although the intensity and hue varied among different pigments. Under blue-and-white glaze conditions, most samples also developed blue coloration, but pigment-dependent differences were more pronounced. In contrast, under unglazed conditions, the colorimetric parameters fluctuated more strongly and the blue appearance was less consistent. These results indicate that the final color of cobalt pigment does not depend solely on the pigment itself, but is closely related to the glaze matrix, firing atmosphere, pigment–glaze contact, and possible elemental migration during firing [10,13,14]. For this study, this finding is particularly significant because it shows that Luanbai glaze does not hinder cobalt-blue coloration; instead, it can provide a favorable glaze environment for cobalt-blue color development under the tested firing conditions.

### 3.3. Refiring Experiments on Authentic Yuan Dynasty Specimens

**Figure 12:**
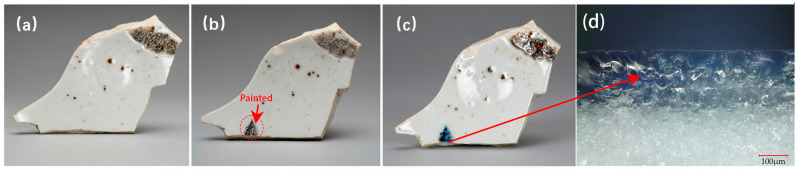
Refiring results for the authentic Yuan Luanbai specimen: (**a**) before painting; (**b**) after painting; (**c**) after firing; (**d**) cross-section showing the interaction between the applied cobalt pigment and the historical glaze layer.

Building upon the simulated firing experiments, authentic Yuan Dynasty Luanbai and blue-and-white sherds were selected for refiring experiments to further evaluate the applicability of the experimental results to historical glaze surfaces. After qh05 cobalt pigment was applied to the Yuan Luanbai sherd and refired at 1300 °C under a reducing atmosphere, the treated area developed cobalt-blue coloration, with average colorimetric values of L* = 32, a* = −6, and b* = −16 (Figure 12). Using the same refiring procedure, the Yuan blue-and-white sherd also developed a blue appearance, with average values of L* = 45, a* = −7, and b* = −13. Both samples showed negative b* values, indicating that qh05 cobalt pigment could develop cobalt-blue coloration on authentic Yuan glaze surfaces under the tested refiring conditions.

Compared with the simulated firing experiments, the refiring experiments are significant because they were conducted directly on authentic Yuan Dynasty glaze surfaces rather than only on laboratory-reconstructed glaze layers. The results provide additional support for the technological feasibility of cobalt-blue coloration on Luanbai glaze. However, this experiment should be understood as a compatibility test rather than a full reconstruction of the original Yuan underglaze-blue production process, because the cobalt pigment was applied to already fired historical glaze surfaces and then refired.

**Figure 13:**
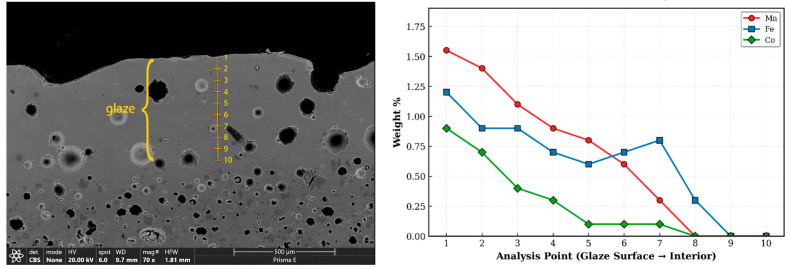
Cross-sectional SEM image and EDS line-scan profiles of the authentic Yuan Luanbai porcelain sherd after application of qh05 cobalt pigment and refiring at 1300 °C in a reducing atmosphere. The numbered points indicate the EDS analysis positions from the glaze surface toward the interior; Mn, Fe, and Co contents are plotted as weight percentages. The colored curves in the EDS profiles represent the elemental contents indicated in the corresponding legends.

The refired Yuan Luanbai specimen exhibited a relatively continuous glaze layer after firing, and the newly applied qh05 cobalt pigment showed clear interaction with the original Luanbai glaze surface (Figure 13). Cross-sectional SEM observation indicates that the original glaze layer remained largely intact after refiring, although local pores and microcracks were observed. The EDS line-scan results show that Mn, Fe, and Co are relatively enriched in the surface or upper part of the glaze layer and then gradually decrease toward the interior. At deeper positions, the contents of Co and Mn fall to very low levels or become nearly undetectable, indicating that the pigment-derived elements are mainly concentrated in the upper glaze/pigment reaction zone. These results suggest that, under the high-temperature reducing refiring conditions used in this study, authentic Yuan Luanbai glaze can support the interaction between qh05 cobalt pigment and the glaze layer and the development of blue coloration. Therefore, the opalescent appearance of Luanbai glaze should not be regarded as an inherent technological barrier to cobalt-blue decoration.

**Figure 14:**
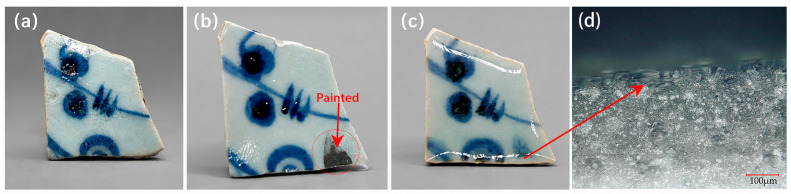
Refiring results for the authentic Yuan blue-and-white specimen: (**a**) before painting; (**b**) after painting; (**c**) after firing; (**d**) cross-section showing the interaction between the applied cobalt pigment and the historical glaze layer.

The corresponding refiring results for the authentic Yuan blue-and-white specimen are shown in Figure 14.

**Figure 15:**
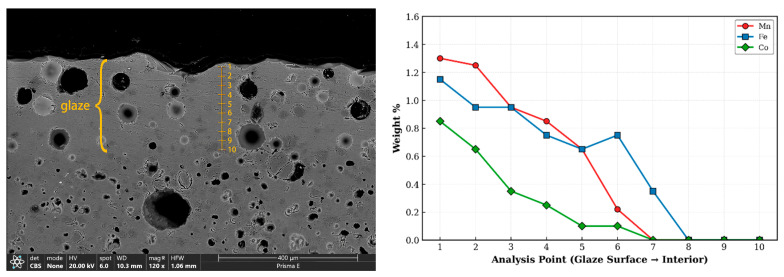
Cross-sectional SEM image and EDS line-scan profiles of the authentic Yuan blue-and-white porcelain sherd after application of qh05 cobalt pigment and refiring at 1300 °C in a reducing atmosphere. The numbered points indicate the EDS analysis positions from the glaze surface toward the interior; Mn, Fe, and Co contents are plotted as weight percentages. The colored curves in the EDS profiles represent the elemental contents indicated in the corresponding legends.

The refired Yuan blue-and-white specimen shows that the newly painted qh05 cobalt pigment also interacted with the original historical glaze after refiring (Figure 15). The SEM cross-section reveals a continuous glaze layer with local pores and microcracks. According to the EDS line-scan results, Mn and Co are relatively enriched in the upper glaze region and decrease gradually toward the inner glaze layer. This elemental gradient is consistent with the partial incorporation or migration of pigment-derived elements into the upper part of the original glaze during refiring. Together with the Luanbai refiring result, this observation further supports that qh05 cobalt pigment can develop blue coloration and interact with authentic Yuan glaze surfaces under the same high-temperature reducing conditions. It should be emphasized that the refiring experiment is intended as a technological compatibility test, rather than a complete reconstruction of the original Yuan blue-and-white production process.

### 3.4. Discussion: Material Basis and Technological Significance of Cobalt-Blue Coloration in Luanbai Glaze

#### 3.4.1. Reconsidering Technical Continuity and Opalescent Appearance

The results of glaze-thickness measurement, body and glaze compositional analysis, and colorimetry suggest that Yuan Dynasty Jingdezhen Luanbai glaze and blue-and-white glaze were not two completely isolated technological systems. Both glaze types have comparable thicknesses of approximately 0.20 mm, and their SiO_2_ and Al_2_O_3_ contents, which form the basic glass network, are also similar. These features indicate that both belong to related high-temperature calcium–alkali glaze traditions in Jingdezhen porcelain production [1,8,24]. Previous archaeometric studies on the relationship between Luanbai and Qingbai glazes from Hutian kiln have also shown that Luanbai glaze developed within the broader Song–Yuan high-fired glaze tradition, rather than as an entirely separate glaze technology [1].

Therefore, the difference between Luanbai and blue-and-white glazes should be understood mainly as an adjustment within a related high-temperature glaze tradition, involving flux proportions, firing conditions, and microstructural development, rather than as a fundamental opposition between two material systems. The opalescent and opaque appearance of Luanbai glaze after firing primarily reflects its optical and microstructural characteristics. It should not be directly equated with an inability of the glaze to soften, interact with cobalt pigment, or support cobalt-blue coloration during firing. In other words, “post-firing transparency” and “in-firing coloration support” are two different issues. The present experimental results indicate that, under the tested high-temperature reducing conditions, Luanbai glaze can support cobalt-blue coloration despite its opalescent appearance.

#### 3.4.2. Combined Effects of Glaze Environment, Pigment Composition, and Microstructure

Previous studies on blue-and-white porcelain have shown that cobalt-blue coloration is not controlled solely by the bulk composition of the cobalt pigment. Instead, it is affected by multiple factors, including the valence state and coordination environment of cobalt, Co-bearing crystalline phases, pigment particle size and distribution, glaze color, the body–glaze interface, and firing atmosphere [13,14,15,28]. Tetrahedrally coordinated Co^2+^ and Co-bearing spinel phases have been identified as important contributors to blue coloration, whereas associated elements such as Mn and Fe may influence the darkness, greyness, and hue of the final color [14,23,29,30]. Studies on the bleeding effect of cobalt pigments further suggest that cobalt migration in vitreous silicate should be understood as a diffusion process, and that crystalline phases such as anorthite or Co-spinel may influence pigment dissolution, diffusion, and color dispersion [10,13].

In the present study, all five cobalt pigments showed blue coloration to varying degrees under Luanbai glaze coverage, whereas the unglazed control group exhibited greater color fluctuations and weaker color stability. This indicates that cobalt pigments have inherent coloration potential, but the development of a relatively consistent underglaze-blue appearance depends strongly on the high-temperature glassy environment provided by the glaze layer. SEM-EDS results show that in both the Luanbai-glazed and blue-and-white-glazed qh05 samples, Co and Mn are mainly enriched in the upper glaze or pigment–glaze interaction region and decrease gradually toward the interior. This surface-to-interior elemental gradient suggests that the cobalt pigment did not remain merely as a surface deposit during firing, but came into contact with, was partially incorporated into, or interacted with the molten or softened glaze layer. By contrast, the unglazed sample shows heterogeneous pigment-particle distribution and lacks a continuous glaze coverage or glassy matrix environment, making it difficult to form a uniform and stable underglaze-blue effect.

It should be emphasized that XPS mainly reflects the near-surface chemical states of the analyzed samples. Therefore, the XPS data should not be used alone as direct proof of the complete pigment–glaze interfacial reaction mechanism. In this study, XPS is treated as complementary evidence for changes in the surface chemical environment, while SEM-EDS line-scan analysis provides cross-sectional information on elemental distribution. Taken together, these two types of evidence support a cautious interpretation: Luanbai glaze coverage is associated with changes in the near-surface chemical environment and cross-sectional distribution of Co, Mn, and Fe, and can provide a favorable glaze environment for cobalt-blue coloration. However, the precise cobalt-bearing phases, valence-state evolution, diffusion depth, and possible formation of specific Co-bearing crystalline phases still require further confirmation by Raman spectroscopy, XAFS, FIB-TEM, or higher-resolution elemental mapping [28,31].

#### 3.4.3. Technological Significance and Limitations of the Refiring Experiments

The refiring experiments provide further support for evaluating the relationship between Luanbai glaze and cobalt-blue coloration on authentic historical glaze surfaces. After qh05 cobalt pigment was applied to the surfaces of authentic Yuan Luanbai and blue-and-white sherds and refired under the same high-temperature reducing conditions, both specimens developed blue coloration. The SEM-EDS results show that pigment-derived elements were mainly concentrated in the upper glaze or pigment–glaze interaction region. This indicates that cobalt pigment can develop blue coloration not only in laboratory-reconstructed Luanbai glaze, but also on authentic Yuan Luanbai glaze surfaces, where it can interact with the original glaze layer. For the central question of this study, this result is significant because it suggests that the opalescent appearance of Luanbai glaze should not be regarded as an inherent technological barrier to cobalt-blue decoration.

Nevertheless, the nature of the refiring experiment should be defined carefully. In this experiment, cobalt pigment was applied to already fired and historically preserved Yuan glaze surfaces and then refired. Therefore, it does not fully reproduce the original one-step production process of Yuan underglaze-blue porcelain. Rather, it should be understood as a technological compatibility test designed to examine whether authentic Yuan Luanbai glaze could support cobalt-blue coloration and pigment–glaze interaction under high-temperature reducing conditions. Accordingly, the conclusion of this study should be expressed as follows: under the experimental conditions used here, Yuan Dynasty Jingdezhen Luanbai glaze can support cobalt-blue coloration and shows technological feasibility as a glaze environment for cobalt-blue decoration, but the experiment does not fully reconstruct or prove the entire original Yuan blue-and-white production process.

Several limitations remain. First, because of the limited number and conservation requirements of archaeological specimens, destructive cross-sectional analysis of authentic Yuan sherds cannot be expanded without restriction. Second, the refiring experiments were mainly represented by qh05 cobalt pigment and therefore cannot cover all possible cobalt pigment types used in the Yuan Dynasty. Third, although SEM-EDS and XPS provide useful information on elemental distribution and near-surface chemical states, they remain limited in determining the exact valence states, coordination environments, and phase identities of Co, Mn, and Fe. Future work should combine Raman spectroscopy, synchrotron-based XAFS, FIB-TEM, EPMA, or high-resolution elemental mapping to further clarify the microscopic mechanism of cobalt-blue coloration in Luanbai glaze [31,32]. Even with these limitations, the present evidence supports the main conclusion that Yuan Dynasty Jingdezhen Luanbai glaze has the technological feasibility to support cobalt-blue coloration under appropriate high-temperature reducing conditions.

## 4. Conclusions

1. Yuan Dynasty Jingdezhen Luanbai glaze and blue-and-white glaze show clear comparability in glaze thickness and basic chemical composition. Both glaze types have an average thickness of approximately 0.20 mm and similar SiO_2_–Al_2_O_3_ contents, suggesting that they belong to related high-temperature calcium–alkali glaze traditions rather than two completely isolated glaze systems.
2. The simulated firing experiments indicate that Luanbai glaze can support cobalt-blue coloration under high-temperature reducing conditions. Compared with the unglazed control samples, the Luanbai-glazed samples showed more consistent blue appearance and clearer pigment–glaze interaction. XPS and SEM-EDS results further show that glaze coverage is associated with changes in the near-surface chemical environment and cross-sectional distribution of Co, Mn, and Fe, indicating that Luanbai glaze can influence the surface chemical state and elemental distribution of cobalt pigments during firing and thereby favor cobalt-blue color development.
3. Refiring experiments on authentic Yuan Dynasty Luanbai and blue-and-white sherds further support the feasibility of cobalt-blue coloration on genuine historical glaze surfaces. These results suggest that the opalescent appearance of Luanbai glaze should not be regarded as an inherent technological barrier to underglaze cobalt decoration. Instead, under the tested firing conditions, Luanbai glaze can provide a favorable glaze environment for cobalt-blue coloration. This study offers experimental evidence for reassessing the technological relationship between Yuan Dynasty Luanbai porcelain and early blue-and-white porcelain in Jingdezhen.

## Figures and Tables

**Table 1:** Main oxide compositions of the experimental Luanbai and blue-and-white glazes (wt.%).

Glaze Type	SiO_2_	Al_2_O_3_	CaO	MgO	K_2_O	Na_2_O	Fe_2_O_3_	TiO_2_	P_2_O_5_
Luanbai glaze	72.71	13.87	5.05	0.21	4.03	2.12	0.89	0.05	0.06
Blue-and-white glaze	71.72	11.98	6.82	0.40	3.92	1.92	1.06	0.07	0.03

**Table 2:** Chemical compositions of the experimental cobalt pigments (wt.%).

Pigment	Na_2_O	MgO	Al_2_O_3_	SiO_2_	MnO	Fe_2_O_3_	CoO
qh01	1.36	13.78	25.77	28.74	18.19	4.69	6.18
qh02	2.91	0.32	33.18	32.29	19.33	6.52	2.51
qh03	1.69	1.35	19.18	41.64	25.53	7.45	1.68
qh04	3.71	0.89	20.59	33.42	26.24	1.11	9.77
qh05	1.42	0.33	19.71	33.6	30.26	2.39	8.26

**Table 3:** Chemical compositions of the glaze layers of Yuan Dynasty Luanbai and blue-and-white porcelains (wt.%).

Sample	SiO_2_	Al_2_O_3_	CaO	MgO	K_2_O	Na_2_O	Fe_2_O_3_	TiO_2_	P_2_O_5_
YLB-665	74.30	13.47	3.18	0.10	3.69	3.34	0.86	0.06	0.09
YLB-666	71.60	13.99	4.78	0.17	2.85	4.80	0.77	0.04	0.05
YLB-667	72.58	14.36	5.07	0.24	3.56	2.19	0.97	0.03	0.03
YLB-669	74.80	12.31	5.15	0.09	2.74	2.84	1.04	0.04	0.03
YLB-673	72.30	14.43	3.38	0.23	3.72	4.25	0.67	0.03	0.09
YLB-676	73.96	12.46	4.36	0.26	5.46	1.56	0.91	0.03	0.03
YLB-678	72.88	12.74	3.07	0.21	3.92	5.33	0.82	0.03	0.03
YLB-679	70.41	14.64	6.42	0.37	3.98	2.44	0.70	0.04	0.04
YLB-685	71.83	12.39	7.22	0.28	3.11	3.05	1.07	0.04	0.03
YQH-8	72.50	12.06	7.29	0.55	4.06	1.43	1.04	0.06	0.04
YQH-34	70.91	12.98	8.10	0.53	3.65	1.45	1.34	0.04	0.05
YQH-176	69.77	13.39	6.17	0.86	4.31	3.40	1.04	0.05	0.05
YQH-185	75.24	11.71	4.37	0.33	3.02	3.44	0.83	0.06	0.02
YQH-295	72.81	13.00	5.33	0.05	3.61	3.14	1.03	0.04	0.03
YQH-297	74.82	11.48	6.04	0.39	3.45	2.01	0.75	0.05	0.01
YQH-300	71.53	12.30	7.39	0.42	3.73	2.76	0.81	0.06	0.04
YQH-304	71.77	13.40	6.72	0.24	2.79	3.31	0.74	0.04	0.04
YQH-321	74.70	12.04	5.27	0.39	2.90	2.71	0.88	0.09	0.07

**Table 4:** Chemical compositions of the bodies of Yuan Dynasty Luanbai and blue-and-white porcelains (wt.%).

Sample	SiO_2_	Al_2_O_3_	CaO	MgO	K_2_O	Na_2_O	Fe_2_O_3_	TiO_2_	P_2_O_5_
YLB-665	73.39	19.98	0.26	0.49	3.08	0.86	0.85	0.09	0.05
YLB-666	65.46	23.64	0.35	1.11	3.39	3.09	1.78	0.19	0.09
YLB-667	64.64	18.83	7.48	0.42	3.53	3.60	0.40	0.10	0.06
YLB-669	80.23	7.75	0.65	0.00	5.27	3.29	2.23	0.15	0.11
YLB-673	74.36	17.55	0.46	0.02	2.51	2.87	1.13	0.10	0.00
YLB-676	70.05	18.59	2.32	0.56	4.88	1.96	0.57	0.06	0.04
YLB-678	72.24	16.08	2.89	0.26	3.33	3.17	0.95	0.06	0.10
YLB-679	67.42	23.69	0.26	0.74	3.58	1.64	1.55	0.12	0.18
YLB-685	67.03	24.89	0.18	0.61	2.92	2.22	1.09	0.05	0.02
YQH-8	60.62	27.23	0.17	1.99	1.36	0.6	6.15	0.89	0.02
YQH-34	70.94	20.44	0.33	1.26	3.02	1.34	1.41	0.25	0.01
YQH-176	69.10	23.91	0.15	0.32	3.34	0.84	1.22	0.11	0.01
YQH-185	69.00	22.59	0.23	0.41	2.85	2.87	0.99	0.06	0.02
YQH-295	68.14	23.77	0.16	0.39	3.21	2.10	1.17	0.05	0.05
YQH-297	61.83	26.62	0.35	1.06	4.41	3.02	1.59	0.12	0.58
YQH-300	69.61	22.69	0.17	0.71	3.56	0.81	1.30	0.15	0.02
YQH-304	69.36	24.12	0.37	0.61	3.30	0.03	1.12	0.09	0.02
YQH-321	68.54	24.18	0.36	0.65	2.75	1.12	1.31	0.08	0.10

**Table 5:** CIELAB colorimetric values of the white-ground areas. L*, a*, and b* indicate lightness, the red–green axis, and the yellow–blue axis, respectively.

Sample	L*	a*	b*
YLB-665	69.61	−2.51	4.16
YLB-666	70.85	−3.26	1.42
YLB-667	70.35	−3.74	2.54
YLB-669	71.91	−2.63	3.51
YLB-673	70.4	−3.34	2.44
YLB-685	75.13	−1.65	1.47
YLB-676	69.98	−3.94	1.92
YLB-678	72.83	−2.4	2.56
YLB-679	62.78	−5.24	2.95
YQH-8	65.56	−3.72	5.14
YQH-34	61.27	−3.53	5.37
YQH-176	72.46	−3.19	5.03
YQH-185	71.15	−4.54	0.17
YQH-297	66.37	−4.13	3.36
YQH-300	66.81	−4.22	2.05
YQH-295	67.32	−4.19	4.53
YQH-304	72.51	−4.02	1.82
YQH-321	68.14	−3.72	1.99

**Table 6:** Average CIELAB colorimetric values of the cobalt-blue motif areas on Yuan blue-and-white specimens. L*, a*, and b* indicate lightness, the red–green axis, and the yellow–blue axis, respectively.

Sample	L*	a*	b*
YQH-8	49	−1	1
YQH-34	28	−4	−1
YQH-176	19	3	−24
YQH-185	21	4	−26
YQH-297	15	2	−14
YQH-300	21	1	−17
YQH-295	12	−2	−4
YQH-304	19	1	−17
YQH-321	18	1	−18

**Table 7:** Average CIELAB colorimetric values of the Luanbai glaze group. L*, a*, and b* indicate lightness, the red–green axis, and the yellow–blue axis, respectively.

No.	L*	a*	b*
qh01	20	7	−23
qh02	34	2	−19
qh03	38	−2	−5
qh04	38	1	−20
qh05	42	3	−24

**Table 8:** Average CIELAB colorimetric values of the blue-and-white glaze group. L*, a*, and b* indicate lightness, the red–green axis, and the yellow–blue axis, respectively.

No.	L*	a*	b*
qh01	4	3	−10
qh02	5	0	−6
qh03	21	−2	12
qh04	5	11	−25
qh05	25	10	−32

**Table 9:** Average CIELAB colorimetric values of the unglazed group. L*, a*, and b* indicate lightness, the red–green axis, and the yellow–blue axis, respectively.

No.	L*	a*	b*
qh01	58	−4	−13
qh02	35	−1	0
qh03	72	4	12
qh04	29	−5	−10
qh05	35	0	−3

**Table 10:** ΔE*ab values between the experimental/refired samples and the archaeological Yuan blue-and-white reference mean.

Group	Mean ΔE*ab from Archaeological Yuan Blue-and-White Reference Mean	Interpretation
Luanbai glaze group	16.4	closer to the reference mean than the unglazed group
Blue-and-white glaze group	21.6	blue coloration, but pigment-dependent variation is clear
Unglazed group	27.1	larger distance from the reference mean and poorer consistency
Refired Luanbai sample	11.9	relatively close to the archaeological reference mean
Refired blue-and-white sample	23.8	blue coloration but with higher lightness difference

## Data Availability

The original contributions presented in this study are included in the article. Further inquiries regarding the data can be directed to the corresponding author.
